# Supplementary material for: Suspected Cases of Chlamydia-Induced Fertility Problems in Sows: What Is the Approach of Austrian Practitioners?
Source: Animals (Basel). 2024 Oct 16;14(20):2983. doi: 10.3390/ani14202983 (PMC11503730; doi:10.3390/ani14202983)
Supplement: Supplementary file 1 [file animals-14-02983-s001.zip › animals-3212859-supplementary.pdf]

Questionnaire (original in german language)

1. In your experience, what is the significance of chlamydial infections in pig farms **in general**?

   1-10 (1=no significance, 10= high significance)

2. In your experience, what is the significance of chlamydia infections in pig farms **in the context of fertility problems**?

   1-10 (1=no significance, 10= high significance)

3. In which form do you observe chlamydia-induced fertility problems?
   - rebreeding
   - infertility
   - abortion
   - mummies
   - weak born piglets/dead-born piglets
   - vaginal discharge
   - others

4. Are you satisfied with the current possibilities in the diagnosis of chlamydia-related fertility disorders?
   - Yes, for me the currently offered tests are sufficient for a diagnosis.
   - No, I cannot provide a proper diagnosis with the tests currently available.
   - Other

5. How often do you run diagnostic tests when you suspect chlamydia-induced fertility disorders in sows?

   1-10 (1=never, 10= in all cases)

6. How do you diagnose 'chlamydia-related fertility issues' within the scope of your capabilities?
   - Antibody testing using complement fixation test (CFT) in serum
   - PCR testing out of vaginal swabs
   - PCR testing out of abortion material
   - PCR testing out of genital tract (e.g. postmortem, slaughterhouse sampling)
   - others

7. How do you proceed therapeutically in the case of suspected or confirmed chlamydial infertility?
   - Use of tetracyclines
   - Use of macrolides
   - use of other antibiotic groups
   - no use of antibiotics
   - others

8. If you use antibiotics in the case of suspected or confirmed chlamydial infertility, what is the usual duration of the treatment?
    - < 5 days
    - 5-10 days
    - 11-15 days
    - 16-21 days
    - At least 21 days
    - I don´t use antibiotics in this case
    - others

9. How do you proceed prophylactically in the case of suspected or confirmed chlamydial infertility? (open question)

10. What suggestions or requests do you have for universities/scientists regarding the improvement of the detection (direct/indirect) of chlamydia in cases of fertility problems? What questions have arisen in your routine practice in this context? (open question)
